# Surgeons agree more on treatment recommendations than on classification of proximal humeral fractures

**DOI:** 10.1186/1471-2474-13-114

**Published:** 2012-06-27

**Authors:** Stig Brorson, Bo Sanderhoff Olsen, Lars Henrik Frich, Steen Lund Jensen, Anne Kathrine Sørensen, Michael Krogsgaard, Asbjørn Hróbjartsson

**Affiliations:** 1 Department of Orthopaedic Surgery, Herlev University Hospital, Herlev Ringvej 75, 2730, Herlev, Denmark; 2 Department of Orthopaedic Surgery, Odense University Hospital, Søndre Boulevard 29, 5000, Odense C, Denmark; 3 Department of Orthopaedic Surgery, Alborg University Hospital, Klinik Farsø, Højgaardsvej 11, 9640, Farsø, Denmark; 4 Department of Orthopaedic Surgery, Bispebjerg University Hospital, Bispebjerg Bakke 23, 2400, Copenhagen NV, Denmark; 5 Nordic Cochrane Centre, Rigshospitalet, Department 3343, Blegdamsvej 9, 2100, Copenhagen Ø, Denmark

## Abstract

**Background:**

Orthopaedic surgeons disagree considerably when classifying fractures of the proximal humerus. However, the clinical implications of low observer agreement remain unclear. The purpose of the study was to compare the agreement on Neer classification with the agreement on treatment recommendations.

**Methods:**

We conducted a multi-centre observer-study. Five experienced shoulder surgeons independently assessed a consecutive series of 193 radiographs at two occasions three months apart. All pairs of radiographs were classified according to Neer. Subsequently, the observers were asked to recommend one of three treatment modalities for each case: non-operative treatment, locking plate osteosynthesis, or hemiarthroplasty.

**Results:**

At both classification rounds mean kappa-values for inter-observer agreement on treatment recommendations (0.48 and 0.52) were significantly higher than the agreement on Neer classification (0.33 and 0.36) (p < 0.001 at both rounds). The highest mean kappa-values were found for inter-observer agreement on non-surgical treatment (0.59 and 0.55). In 36% (345 out of 965) of observations an observer changed Neer category between first and second classification round. However, in only 34% of these cases (116 out of 345) the observers changed their treatment recommendations.

**Conclusions:**

We found a significantly higher agreement on treatment recommendations compared to agreement on fracture classification. The low observer agreement on the Neer classification reported in several observer studies may have less clinical importance than previously assumed. However, inter-observer agreement did not exceed moderate levels.

## Background

Orthopaedic surgeons disagree considerably when classifying fractures of the proximal humerus according to the Neer classification [1]. However, the clinical implications of low observer agreement on fracture classification remain unclear. Inter-observer agreement on binary treatment decision has been reported [2] but to our knowledge, no study has compared the agreement on classification with the agreement on treatment recommendations.

The purpose of the study was to compare the agreement on Neer classification with the agreement on treatment recommendations using kappa-statistics. Our secondary aim was to study the impact of information on age on the agreement on treatment recommendations.

We conducted a multi-centre observer-study among experienced shoulder surgeons assessing a large consecutive series of unselected radiographs of proximal humeral fractures.

## Methods

Within an arbitrarily chosen period of two months (October and November 2007) all patients discharged from five Danish university hospitals (orthopaedic ward or emergency unit) diagnosed with a fracture of the proximal humerus were identified. Imaging material was collected, stored, and presented electronically by the first author who was not serving as an observer. Plain anterior-posterior and scapular-lateral radiographs should be available for each case. We excluded pathological fractures, humeral shaft fractures, healed fractures, fractures in skeletal immature, pseudoarthroses, and miscoded cases. No further selection of images was allowed.

Five shoulder fellowship trained surgeons (BO, AS, LF, MK, SJ) served as observers. They were not informed about the purpose and design of the study. The observers were blinded to the identity of the patients, institutions, and the treatments given.

The five observers independently assessed and classified all sets of radiographs on two occasions three months apart. The observers were allowed to use a goniometer, a numbered diagram of the original 16-category Neer classification [1], and a written definition of displacement. There was no time limit. First, the observers were asked to assess whether the quality of imaging material was sufficient for classification and treatment purposes in each case. Second, all pairs of radiographs were classified according to the Neer classification. Third, the observers were asked to recommend one of three treatment modalities for each case: non-operative treatment, locking plate osteosynthesis, or hemiarthroplasty.

Three months later the observers independently re-assessed and re-classified all sets of radiographs in a new, random order. At this classification round the observers were additionally provided with information on the patient’s age.

### Statistical methods

Mean kappa-values for inter-observer agreement and ninety-five percent confidence intervals were calculated. Kappa-values were interpreted qualitatively according to Landis and Koch [3]: kappa-values less than 0 indicate poor agreement, 0.00-0.20 slight agreement, 0.21-0.40 fair agreement, 0.41-0.60 moderate agreement, 0.61-0.80 substantial agreement, and 0.81-1.00 excellent agreement.

For both classification rounds mean kappa-values and ninety-five percent confidence intervals were calculated for inter-observer agreement on 1) adequacy of radiographs for classification and treatment purposes, 2) classification according to the 16-category Neer classification, and 3) treatment recommendations: non-operative, locking plate osteosynthesis, or hemiarthroplasty.

Changes in mean kappa-values for inter-observer agreement on classification and treatment recommendations between first and second classification round were analyzed. The statistical significance of observed differences in mean kappa-values was calculated using a bootstrapping technique.

For all cases of change in classification category from first to second round we recorded if the change in classification was accompanied by a change in treatment recommendation.

Finally, we conducted a sensitivity analysis by omitting the most extreme observer and repeating the calculations.

STATA, version 11.0 was used for calculation of kappa statistics and confidence intervals (StataCorp, 2009, Collage Station, Texas, USA). R statistical software version 2.12.1 ‘bootstrap’ package was used for bootstrapping (R Foundation for statistical software, 2010, Vienna, Austria).

## Results

No kappa-values exceeded ‘moderate agreement’ (Table 1) as defined by Landis and Koch [3]. The highest kappa values were found for inter-observer agreement on non-surgical treatment on both classification rounds, mean kappa 0.59 (95% CI 0.52-0.66) and 0.55 (95% CI 0.48-0.63). In 28 cases four out of five observers disagreed on the Neer classification at either first or second round. Total unanimity was attained in only 24 cases at either first or second round (11 minimally displaced fractures, six surgical neck fractures, and seven four-part fractures).

**Table 1:** Mean kappa-values and ninety-five percent confidence intervals for inter-observer agreement between five observers

	Neer classification	Treatment recommendations	Adequacy of images
First round	0.33 (0.29-0.38)	0.48 (0.43-0.54)	0.29 (0.22-0.38)
		0.59^1^ (0.52-0.66)	
		0.38^2^ (0.31-0.46)	
		0.42^3^ (0.35-0.49)	
Second round^4^	0.36 (0.31-0.42)	0.52 (0.45-0.58)	0.24 (0.18-0.31)
		0.55^1^ (0.48-0.63)	
		0.53^2^ (0.44-0.61)	
		0.42^3^ (0.34-0.49)	

^1^ Non-surgical treatment.

^2^ Locking plate osteosynthesis.

^3^ Hemiarthroplasty.

^4^ Patients age known to observers.

The mean kappa-values for inter-observer agreement on adequacy of images at the two rounds were lower than kappa-values for agreement on treatment recommendations (difference 0.19 and 0.28; p < 0.001 at both rounds).

In 36% (345 out of 965) of observations an observer changed Neer category between first and second classification round. However, in only 34% of these cases (116 out of 345) the observers changed their treatment recommendations.

At both rounds mean kappa-values for inter-observer agreement on treatment recommendations were significantly higher than the agreement on Neer classification (differences 0.15 and 0.16; p < 0.001 at both rounds).

At the second round, when information on the patients’ age was added, mean kappa-values for inter-observer agreement on classification and treatment increased slightly. The differences were not statistically significant (p = 0.45 and p = 0.36). However, the mean kappa-values for inter-observer agreement on recommending locking plate osteosynthesis improved significantly from first to second round (difference 0.15; p = 0.012).

By adding a sensitivity analysis excluding the most ‘extreme’ observer only a slight and statistically non-significant improvement of mean kappa-values for inter-observer agreement on classification and treatment recommendations was found.

## Discussion

Shoulder surgeons agree significantly more on treatment decisions, mean kappa-values 0.48 (95% CI 0.43-0.54) and 0.52 (95% CI 0.45-0.58), as compared to fracture classification, mean kappa-values 0.33 (95% CI 0.29-0.38) and 0.36 (95% CI 0.31-0.42). Nonetheless, the agreement is still not better than moderate. Access to information on the patients’ age did not improve inter-observer agreement importantly. In 36% of observations an observer changed Neer category between first and second classification round. However, only 34% of these changes were followed by a change in treatment recommendation.

### Strengths and weaknesses of the study

The strength of our study is the large, consecutive and unselected series of imaging material. All observers were experienced shoulder surgeons from different shoulder units blinded to the hypothesis and design of the study. The design of the study enabled identification of potential changes in inter-observer agreement by adding information of the patients’ age.

The weakness of our study is the exclusive use of senior shoulder surgeons as observers. Our results should be extrapolated with caution to a less experienced population of surgeons. The clinical choice of treatment modality may be affected by several other factors than the fracture appearance on radiographs, e.g. information on comorbidity, bone quality, or functional status of the patient. On the one hand, a systematic review of observer studies [4] did not find that observers disagree on the Neer classification because of differences in clinical experience. On the other hand, experienced observers seem to respond better on training in classification [5].

### Previous studies

In Bernstein et al. [2] two orthopaedic residents and two attending shoulder surgeons classified 20 cases and suggested operative or non-operative management based on plain axillary and anteroposterior radiographs. Overall kappa-value for inter-observer agreement on a 16-category Neer classification was 0.52 (CI not reported). Inter-observer agreement for binary decision to treat operatively was 0.65 (CI not reported). The differences in kappa-values were not compared or tested statistically. We also found the highest level of inter-observer agreement in decisions to treat operatively, mean kappa-values 0.59 (95% CI 0.52-0.66) and 0.55 (95% CI 0.48-0.63), but we did not find kappa-values exceeding moderate agreement.

In Petit et al. [6] 8 orthopaedic surgeons assessed 32 operative cases and 6 randomly chosen non-operative cases of proximal humeral fractures and suggested one out of six treatment modalities. Thus, the ‘prevalence’ of displaced fractures was much higher than in an unselected material. Each case presentation included the pertinent history, physical examination findings, and medical comorbidities along with at least 3 radiographs. Overall weighted kappa for inter-observer agreement on treatment recommendation was 0.41 (95% CI 0.38-0.44, weights not reported). Agreement of treatment recommendation was not compared to agreement on classification. If the number of treatment modalities was reduced to three (non-surgical, internal fixation, hemiarthroplasty) a non-significant increase from 0.41 (95% CI 0.38-0.44) to 0.45 (95% CI 0.42-0.48) was observed. No significant difference between levels of experience was found and no weighted kappa-value exceeded moderate level.

A study on the Garden classification for fractures of the femoral neck [7] reported that 69% of changes in classification were not followed by any change in treatment recommendation. They found that information on age plays a critical role in determining if an observer’s change in classification is followed by change in treatment recommendation. Similarly, we found that only 34% of the changes in classification were followed by a change in treatment recommendation. However, we only found a small and statistically non-significant increase in inter-observer agreement after adding information on the patients’ age.

### Kappa and the distribution of categories

The value of kappa depends on the marginal distribution of the categories studied, that is, the ‘prevalence’ of each Neer category [8,9] High kappa-values are harder to obtain if the ‘prevalence’ of the categories is very low or very high. Thus, measures of agreement cannot be compared across populations with different ‘prevalences’ of the categories under study.

The proportion of Neer categories (a surrogate measure for prevalence) in our unselected population differed from previous studies. I his classical article from 1970 Neer reported a proportion of non-displaced fractures of 85%. In a large epidemiological study Court-Brown et al. [10] reported a proportion of non-displaced fractures of 49% compared to 33% and 29% in our study. Different age distributions may affect the proportion of categories. The mean age in Neer’s study was 55.6 years (range 22–89), 66 years (range 13–98) in Court-Brown’s study, and 67.2 (range 17–98) in our study.

### Conceptions of displacement

We found a higher proportion of four-part fractures than previously reported (17% and 18% compared to 2-10% reported elsewhere) [10,11]. The difference may be ascribed to different conceptions of displacement. In three- and four-part fractures it is not clearly defined whether all involved segments should be displaced according to Neer’s definition. Different approaches of the observers in our study may explain the markedly differences in proportion of four-part fractures (range 8.3% to 34.7%) and the relatively low inter-observer agreement on four-part fractures, mean kappa 0.39 (95% CI 0.28-0.49) and 0.41 (95% CI 0.30-0.51). Such differences in approach may lead to inconsistencies in the interpretation of data in the scientific literature and in the conduct of clinical studies and systematic reviews.

### The patient’s age and observer agreement

No evidence-based treatment recommendations based on patients’ age are available. For example, it is not clear whether osteosynthesis with locking plate is an option in the very elderly, or whether hemiarthroplasty is an option in younger patients with a life expectancy exceeding the expected survival of the prosthesis. By adding information on the patients’ age a slight but non-significant increase in mean kappa-values for inter-observer agreement on classification and treatment was found. Post hoc we observed a statistically significant increase in kappa-value from 0.38 (95% CI 0.31-0.46) to 0.53 (95% CI 0.44-0.61) in inter-observer agreement on the use of locking plates after adding information on the patients’ age (p = 0.012). An age-sensitive decision on locking plate osteosynthesis may reflect an assumption on an upper age limit for this treatment.

### Implications for clinical care and research

We found a significantly higher inter-observer agreement on treatment decisions compared to agreement on fracture classification. This may have clinical implications. If the choice of classification category only influences treatment decisions to a limited degree the poor agreement on fracture classification reported previously [4,12,13] may be less problematic. However, kappa-values below 0.60 are still unsatisfactory from a clinical perspective.

Future studies may address the changes in inter-observer agreement on treatment recommendations by adding information on comorbidity, or by adding the entire medical record. Prospective observer-studies, where surgeons independently decide on treatment based on all relevant information in real time, may contribute to elucidate the sequence of decisions in the assessment of proximal humeral fractures, and hopefully isolate important reasons for disagreement.

## Conclusions

In summary, we found a significantly higher inter-observer agreement on treatment decisions compared to agreement on fracture classification. The low observer agreement on the Neer classification reported in numerous observer studies may have less clinical importance than previously assumed.

## Ethical approval

The study did not involve human individuals and ethical approval was waived by The Regional Committees on Health Research Ethics of the Capital Region of Denmark (H-3-2012-FSP35).

## Competing interests

The authors declare that they have no competing interests. SB was supported financially by Göran Bauer’s Grant, The Danish Rheumatism Association, and the Danish Agency for Science, Technology, and Innovation.

## Authors’ contributions

SB had the idea and designed the study. SB, AS, LF, MK, and SJ gathered the radiographs. BO, AS, LF, MK, and SJ classified the cases. Statistical analysis was conducted by SB and AH. SB wrote the manuscript. All authors were involved in critical revision of the paper. All authors read and approved the final manuscript.

## Pre-publication history

The pre-publication history for this paper can be accessed here:

http://www.biomedcentral.com/1471-2474/13/114/prepub
